# Supplementary material for: Evaluation of Selective COX-2 Inhibition and In Silico Study of Kuwanon Derivatives Isolated from Morus alba
Source: Int J Mol Sci. 2021 Apr 1;22(7):3659. doi: 10.3390/ijms22073659 (PMC8036738; doi:10.3390/ijms22073659)
Supplement: Supplementary file 1 [file ijms-22-03659-s001.pdf]

Supplementary data

Evaluation of selective COX-2 inhibition and *in silico* study of kuwanon derivatives isolated from *Morus alba*

Seung-Hwa Baek[1,2†], Sungbo Hwang[1†], Tamina Park[1,3], Yoon-Ju Kwon[4], Myounglae Cho[4], and Daeui Park [1,2,3,*]

[1] Department of Predictive Toxicology, Korea Institute of Toxicology, Daejeon 34114, South Korea; seunghwa.beak@kitox.re.kr (S.H.B.); sungbo.hwang@kitox.re.kr (S.H.); tamina.park@kitox.re.kr(T.P.)
[2] Center for Convergent Research of Emerging Virus Infection, Korea Research Institute of Chemical Technology, Daejeon 34114, South Korea
[3] Department of Human and Environmental Toxicology, University of Science and Technology, Daejeon 34113, South Korea
[4] National Institute for Korean Medicine Development, Gyeongsan 38540, Republic of Korea; XXX@nikom.or.kr (Y.J.K.); meanglae@nikom.or.kr (M.C.)

† Equally contributed to this work
* Correspondence: daeui.park@kitox.re.kr; Tel.: +82-42-610-8844 (D.P.)

Supplemental methods

Definition of binding site

It is important to define a pocket site in the target protein to perform docking simulation and FMO calculation. For docking and FMO calculation of kuwanon derivatives, we used the X-ray crystal structure of human COX-2 (PDB ID: 5IKR), not the homology modeling structure. The crystal structure has a high resolution as 2.43 Å. However, there was no x-ray crystal structure of human COX-2 bound to chemocoxib A (positive control) to define a pocket site. Therefore, we used the x-ray crystal structure of murine COX-2 (PDB ID: 4OTJ, 2.11Å resolution) complexed with chemocoxib A to define the pocket site of COX-2. Since the murine COX-2 crystal structure was very similar to the human COX-2 crystal structure. we found the five key residues in the pocket site of murine COX-2, which consists of gate and membrane-binding domain, using FMO calculation and PIEDA. Among the key residues, the four residues were the same amino acids between murine and human COX-2, but the Tyr122 residue in murine COX-2 was His122 in human COX-2. Therefore, we performed to compare the interaction energy between Tyr122(murine COX-2)-chemocoxib A and His122(human COX-2)-kuwanon derivatives.

Conformational evaluation of binding poses for each kuwanon derivatives

To apply high conformational variability of inhibitors during docking simulation, we performed the molecular docking simulations about 20 poses of inhibitors using AutoDock Vina program. AutoDock Vina has been applied to ligand-flexible docking. Ligand-flexible docking is regarded as high conformational change of inhibitors during docking simulation. We attached each binding score and the RMSDs of conformation about inhibitor (Table S1). In addition, we visualized all binding poses about inhibitors (Figure S6). As a result. the binding poses for each kuwanon derivative were distributed in a wide membrane-binding domain. In addition, we performed to superimpose between best binding pose and other poses for each kuwanon derivatives (Figure S7). The average RMSD

between best binding pose and other poses for all kuwanon derivatives are 9.303 Å in kuwanon A, 10.651 Å in kuwanon B, 7.313 Å in kuwanon C, 7.956 Å in kuwanon E, and 11.757 Å in kuwanon H. Therefore, our approach considers not only conformational variability but also location variability using AutoDock Vina. In addition, FMO calculation and PIEDA were calculated based on 20 ranking poses for each kuwanon derivative, and the structures were calculated to lowest interaction energy between COX-2 and kuwanon derivatives were selected.

Conformational variability analysis of compound converted 2D to 3D structure

The initial molecular structure is important to determine the binding pose calculated in molecular docking simulation. To consider the importance of initial structure, we download the chemical structure used in Pubchem. Pubchem has generated and owned a 3D structure which bioactivity can be considered. [J Cheminform 5, 1 (2013)]. The 3D structures of kuwanon A/B/C/E can be downloaded in Pubchem. However, we can download only 2D structures of kuwanon H from Pubchem database. To confirm whether conformational variability is maintained even with energy minimization, we perform molecular docking simulation for kuwanon A with 3D structure generated using 2D Pubchem structure. First, we downloaded 2D structure of Kuwanon A from Pubchem database (https://pubmed.ncbi.nlm.nih.gov/). Second, the 3D structure of kuwanon A was generated by Chem3D Ultra 7.0 program in ChemOffice package with energy minimization. Chem3D Ultra 7.0 program contains force field-based energy minimization using Molecular Mechanics 2 (MM2) force field [J. Am. Chem. Soc. 1977, 99, 25, 8127–8134]. Last, we perform molecular docking simulation with AutoDock Vina for kuwanon A generated using energy minimization. The result of molecular docking simulation was described in Table S3.

The rank of docking score between Pubchem and generated 3D structure was highly correlated each other, which range of docking score using 3D structure in Pubchem was calculated from -8.9 to -6.8 kcal/mol but that using generated 3D structure was calculated from -8.1 to -6.2 kcal/mol. The

spearman correlation value based on docking score was calculated to 0.991 (p-value < 2.2e-16). All binding poses and superimposed structure for kuwanon A generated 3D structure using 2D Pubchem structure were described in Figure S8.

The binding poses are generated similar to the distribution of kuwanon A with 3D structure downloaded in Pubchem (Figure S8-a). To determine conformational variability numerically, all binding poses were superimposed and the RMSD was calculated (Figure S8-b). The average RMSD based on best binding pose was calculated to 11.074 Å compared to 9.303 Å for kuwanon A with 3D structure downloaded in Pubchem. The all kuwanon A with 3D structure using ChemOffice and Pubchem was superimposed and the average RMSD is calculated to 12.793 Å. Therefore, the conformation variability is maintained although the 3D structure is generated by ChemOffice and we have used kuwanon H, which is generated 3D structure using ChemOffice, in molecular docking simulation.

Supplementary Figures

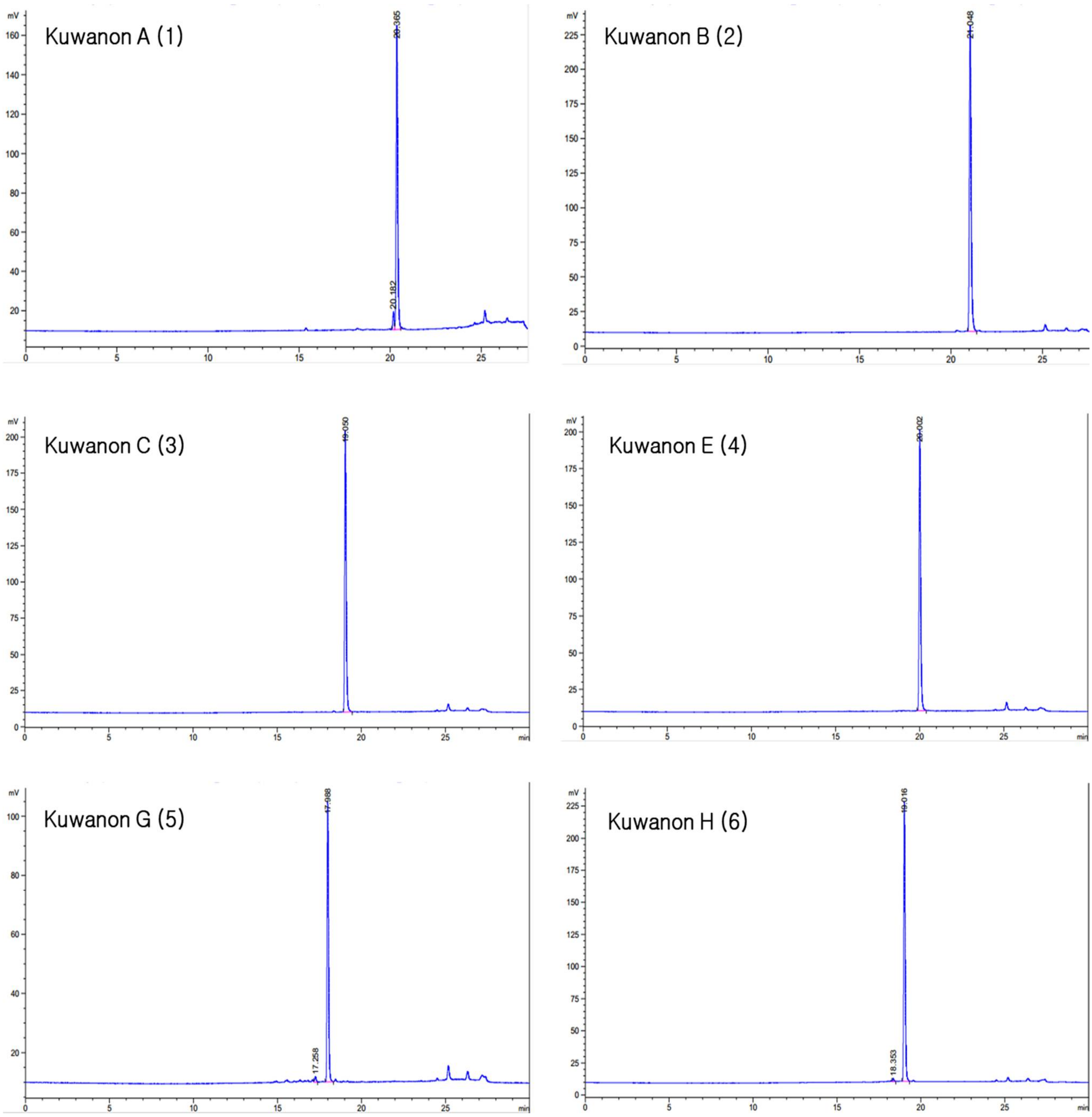

Fig. S1. HPLC profiles of kuwanon derivatives. Kuwanon A (1) (20.365 min), kuwanon B (2) (21.048 min), kuwanon C (3) (19.050 min), kuwanon E (4) (20.002 min), kuwanon G (5) (17.988 min), and kuwanon H (6) (19.016 min).

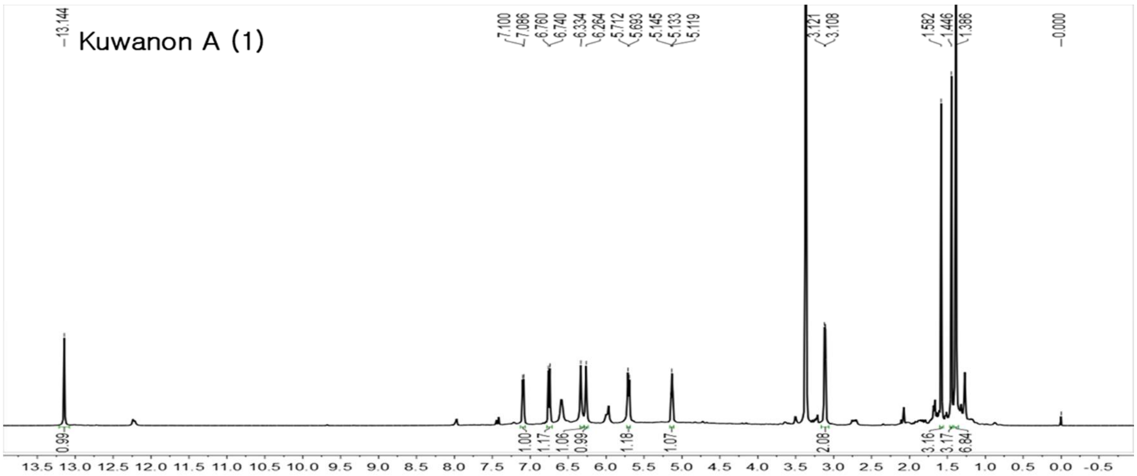
Kuwanon A (1)
13.144
7.100
7.086
6.760
6.740
6.334
6.264
5.712
5.693
5.145
5.133
5.119
3.121
3.108
1.582
1.446
1.386
0.000
0.99
1.00
1.17
1.06
0.99
1.18
1.07
2.08
3.16
3.17
6.84

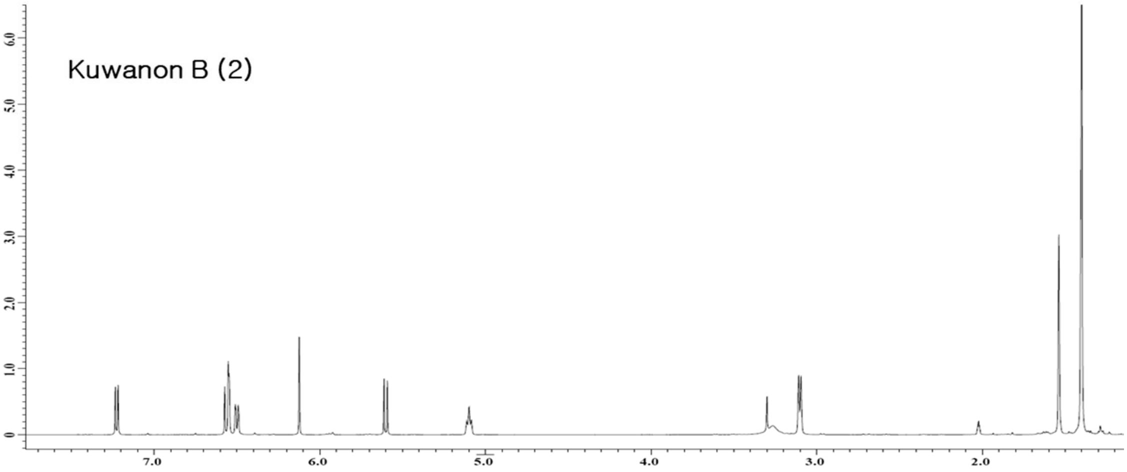
Kuwanon B (2)

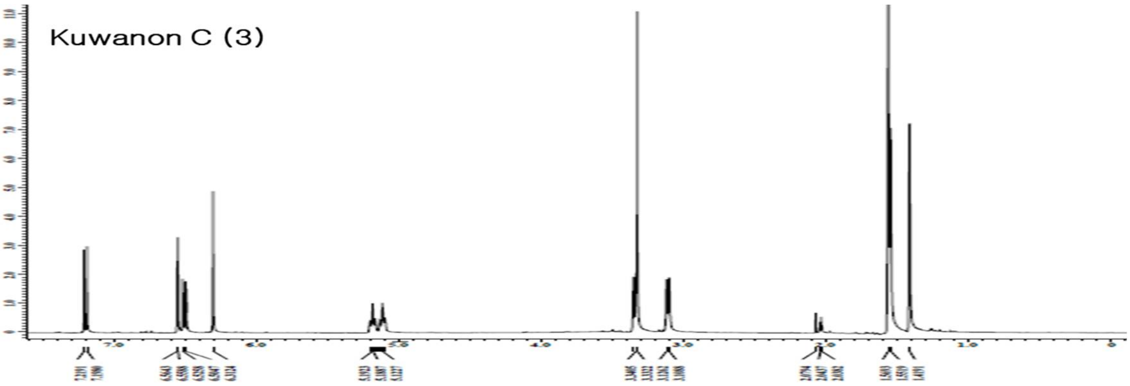
Kuwanon C (3)

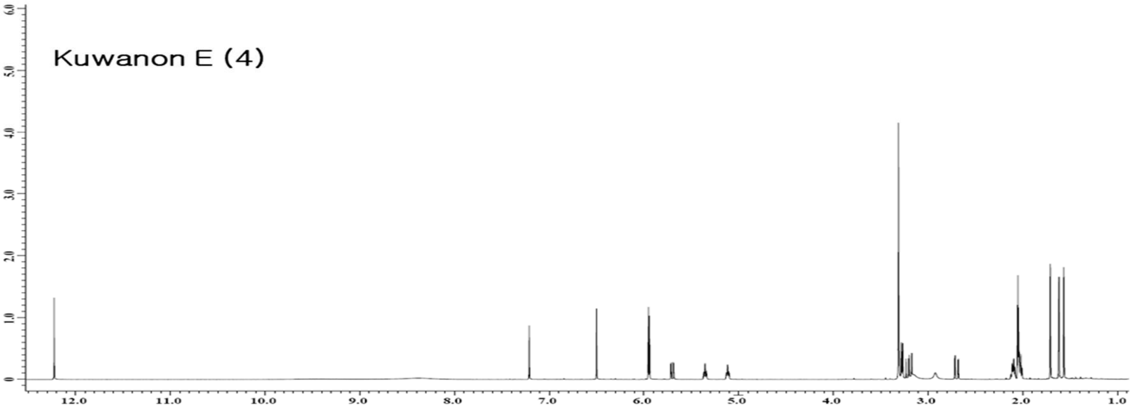
Kuwanon E (4)

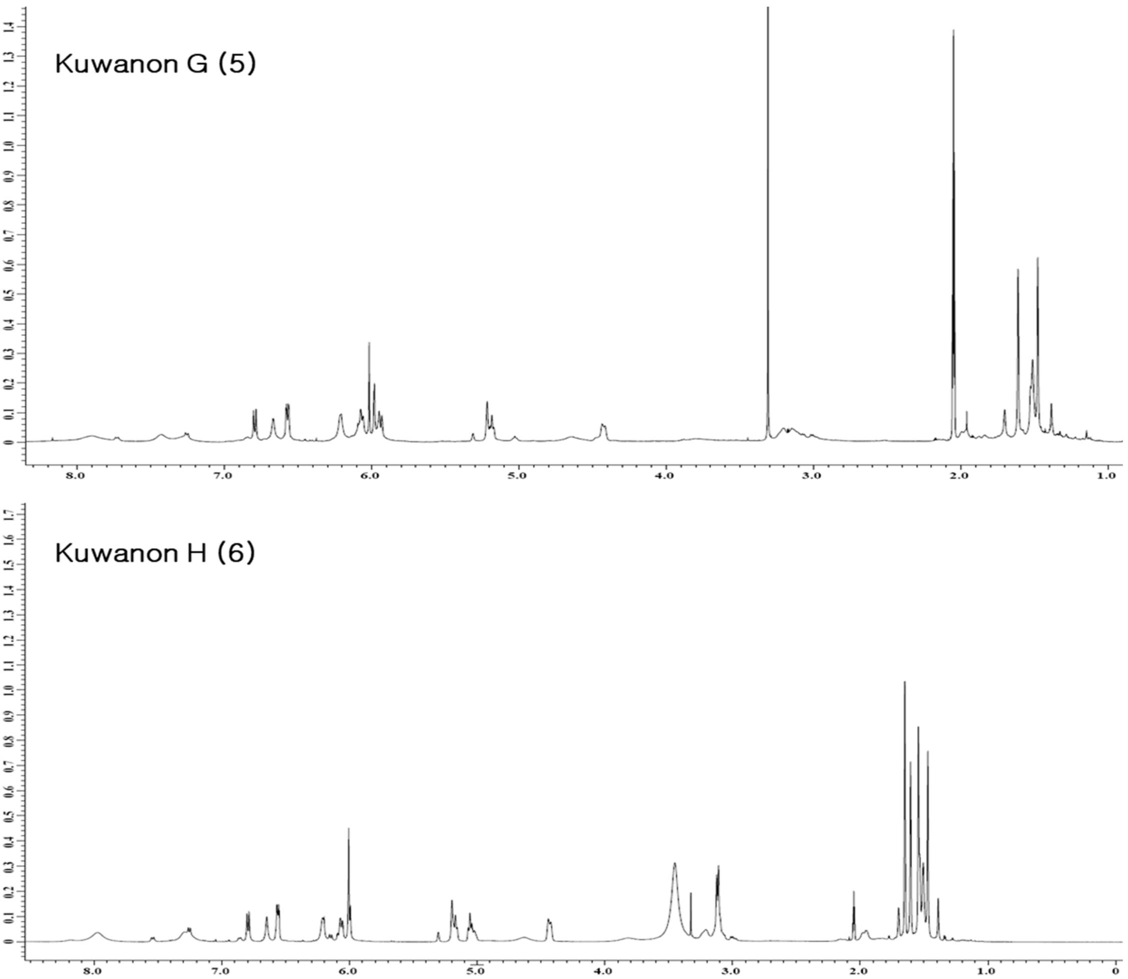

Fig. S2. ^{1}H-NMR spectrum of kuwanon derivatives (in Acetone-d_6, 500 MHz).

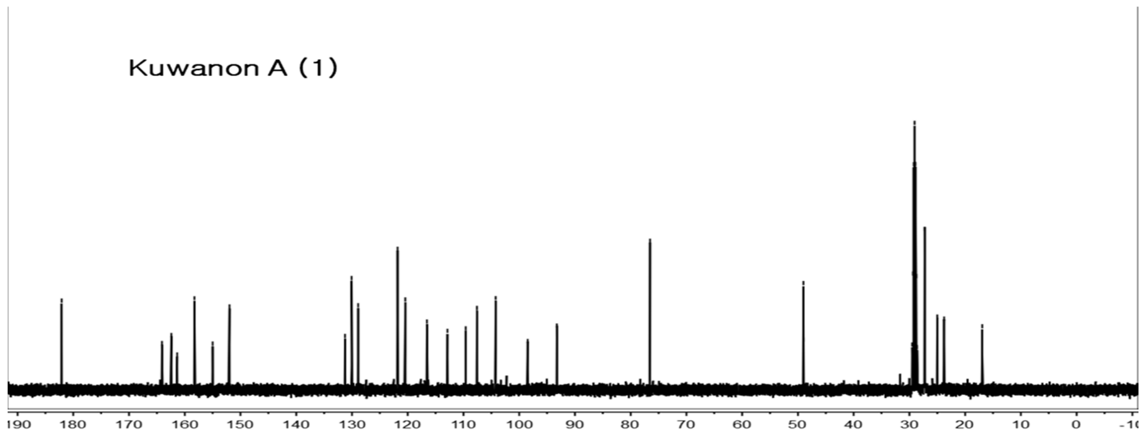
Kuwanon A (1)

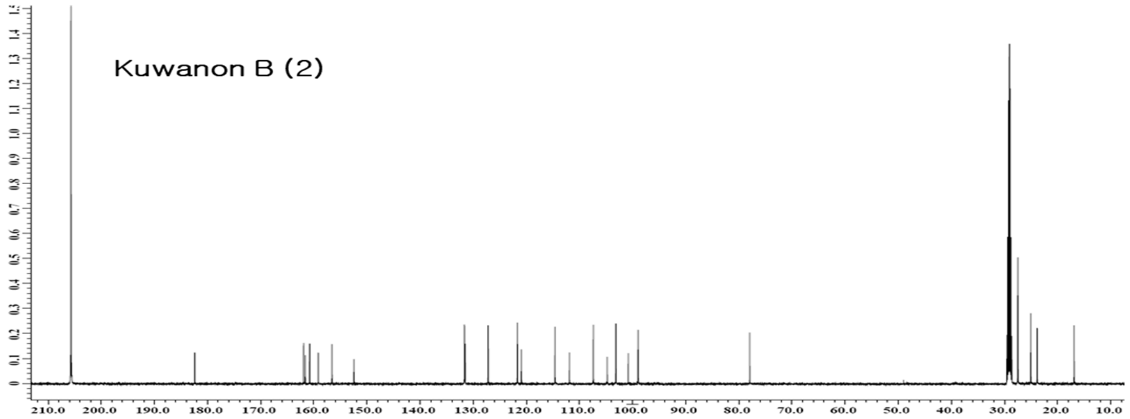
Kuwanon B (2)

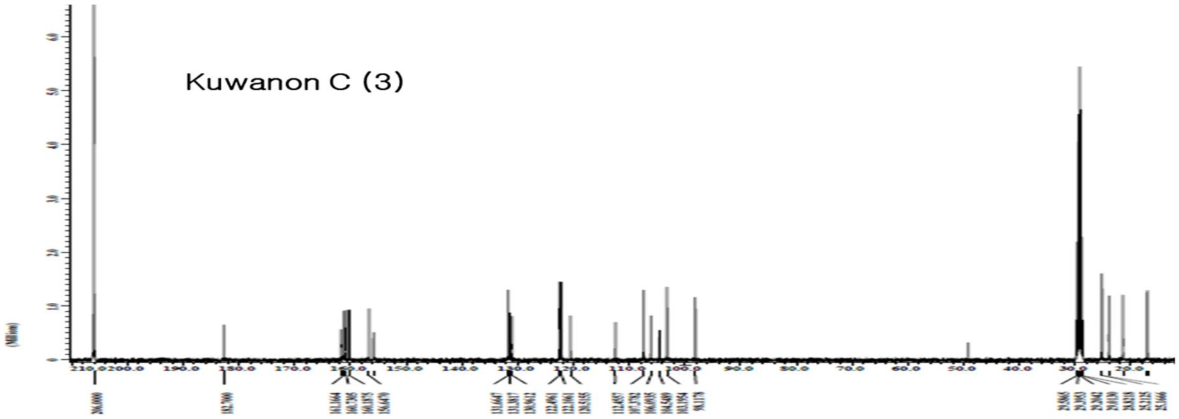
Kuwanon C (3)

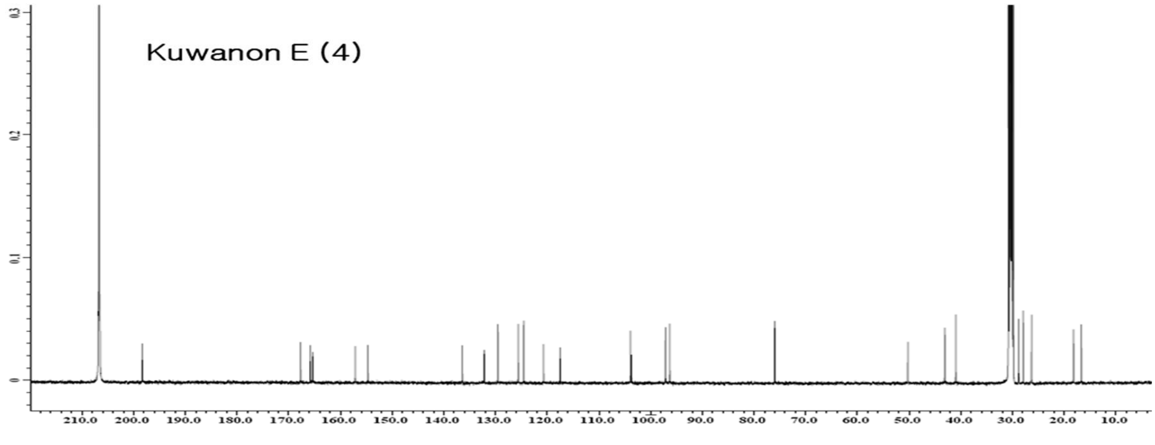
Kuwanon E (4)

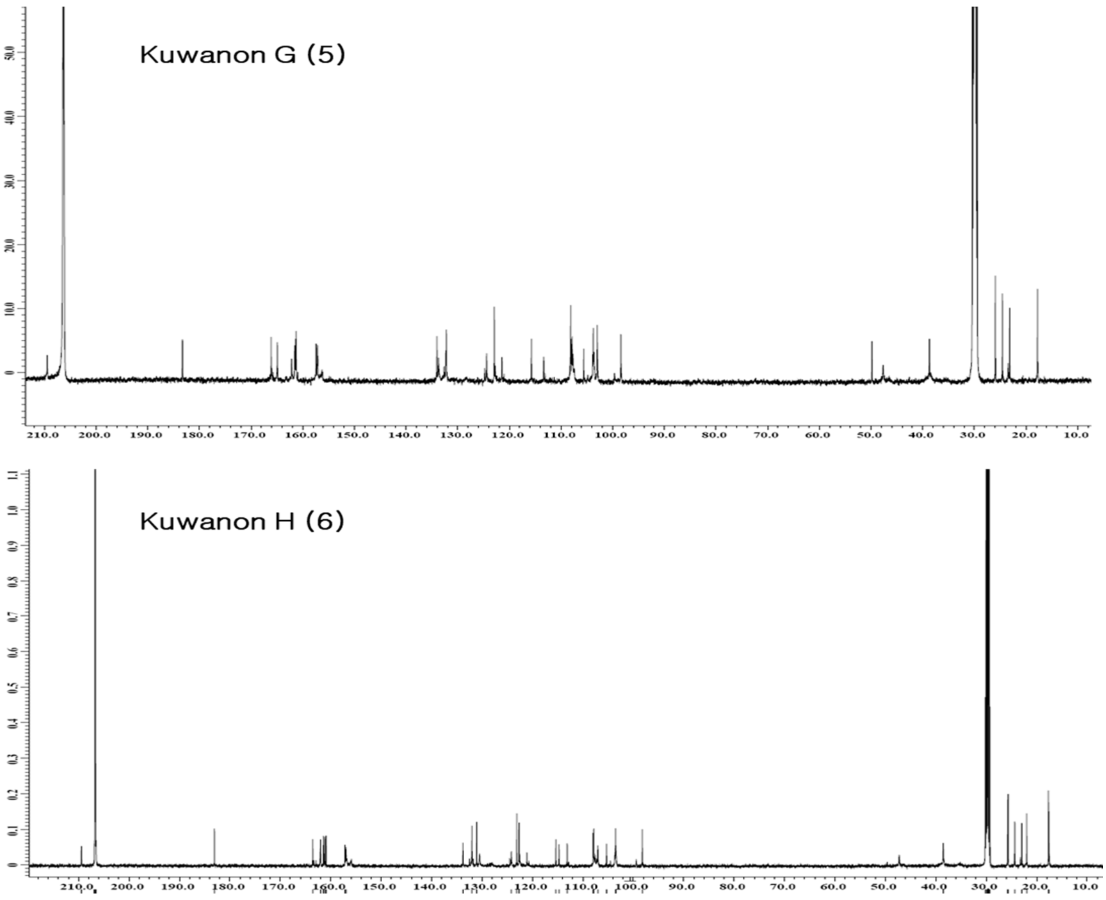

Fig. S3. ^{13}C-NMR spectrum of kuwanon derivatives (in Acetone-d_6, 125 MHz).

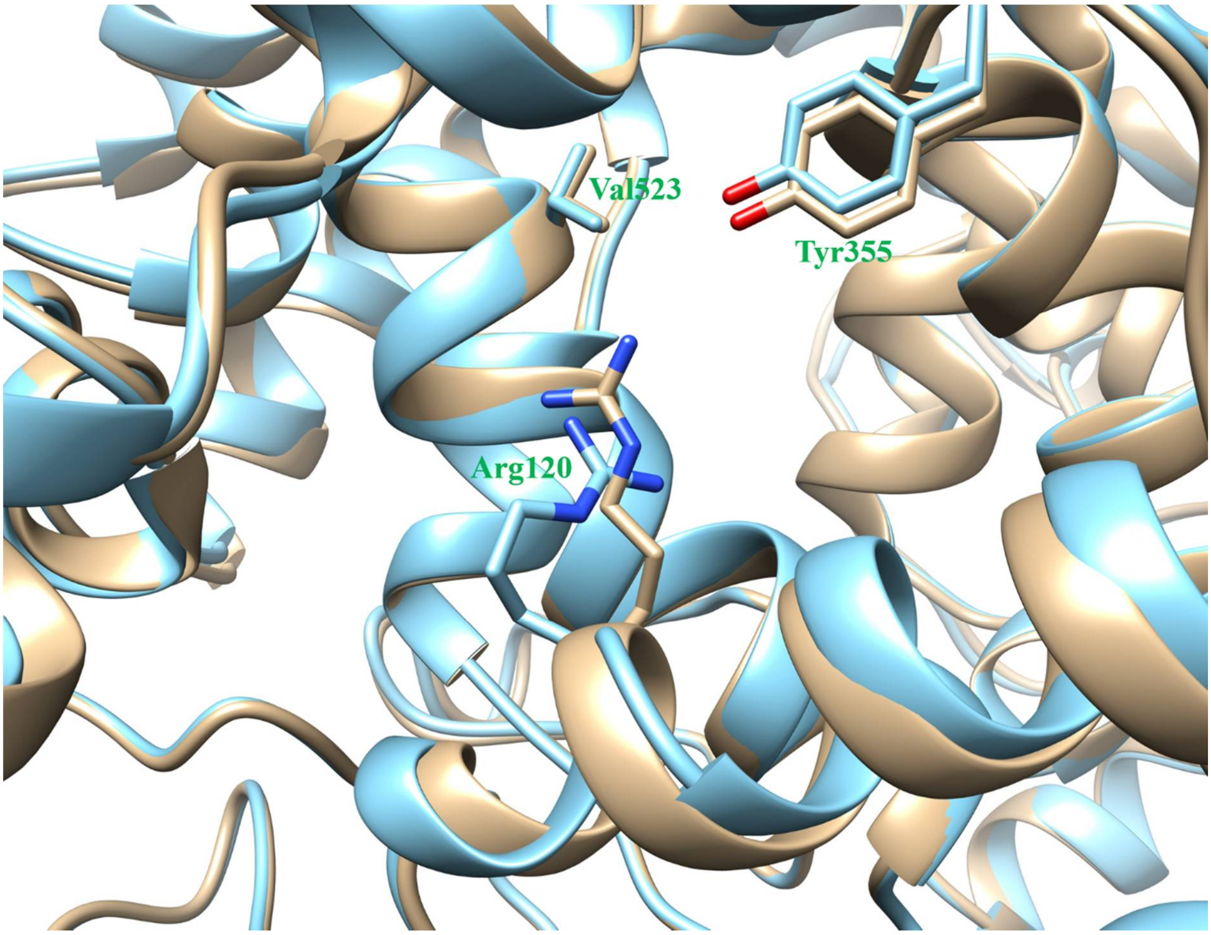

Fig. S4. Superimposed COX-2 structure between mouse (PDB ID: 4OTJ) and human (PDB ID: 5IKR). Tyr355 and Val523 are same conformation but Arg120 is different conformation. Arg120 conformation of mouse COX-2 is located behind Arg120 conformation of human COX-2.

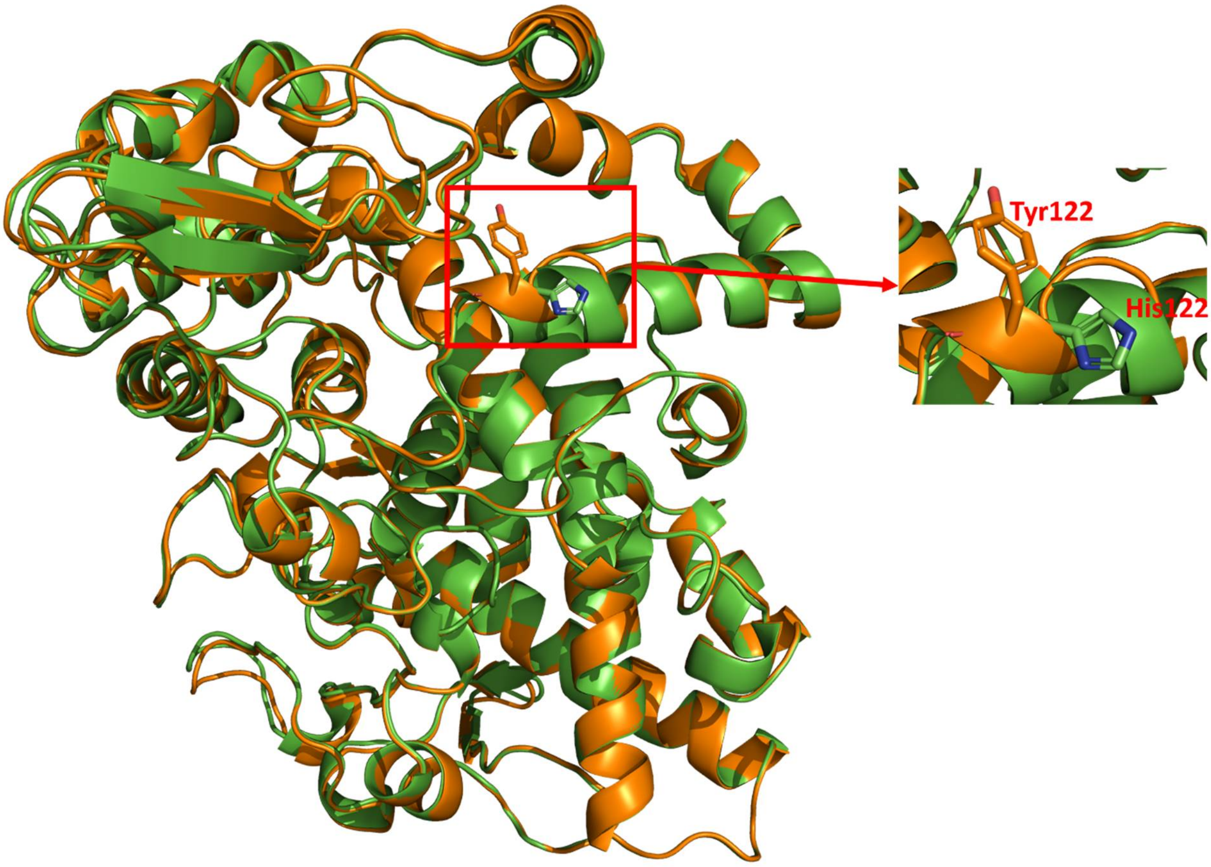

Fig. S5. Superimposed COX-2 structure between mouse (PDB ID: 4OTJ) and human (PDB ID: 5IKR).

Tyr122 and His122 are the same locations in two COX-2 proteins.

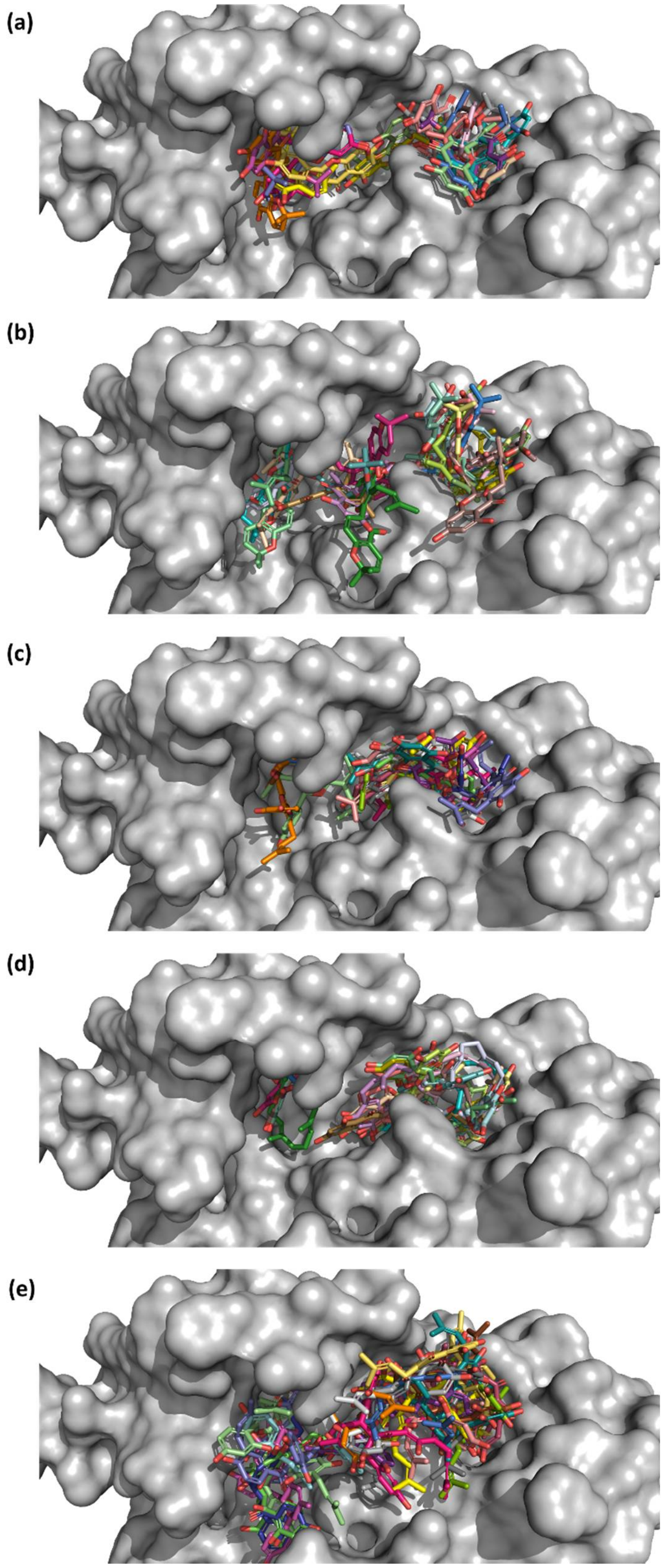

Figure S6. All binding pose for (a) Kuwanon A, (b) Kuwanon B, (c) Kuwanon C, (d) Kuwanon E, and (e) Kuwanon H. The binding poses were generated by molecular docking simulation using AutoDock Vina. The number of binding poses for each Kuwanon derivative was 20. The binding poses were distributed in a wide membrane domain.

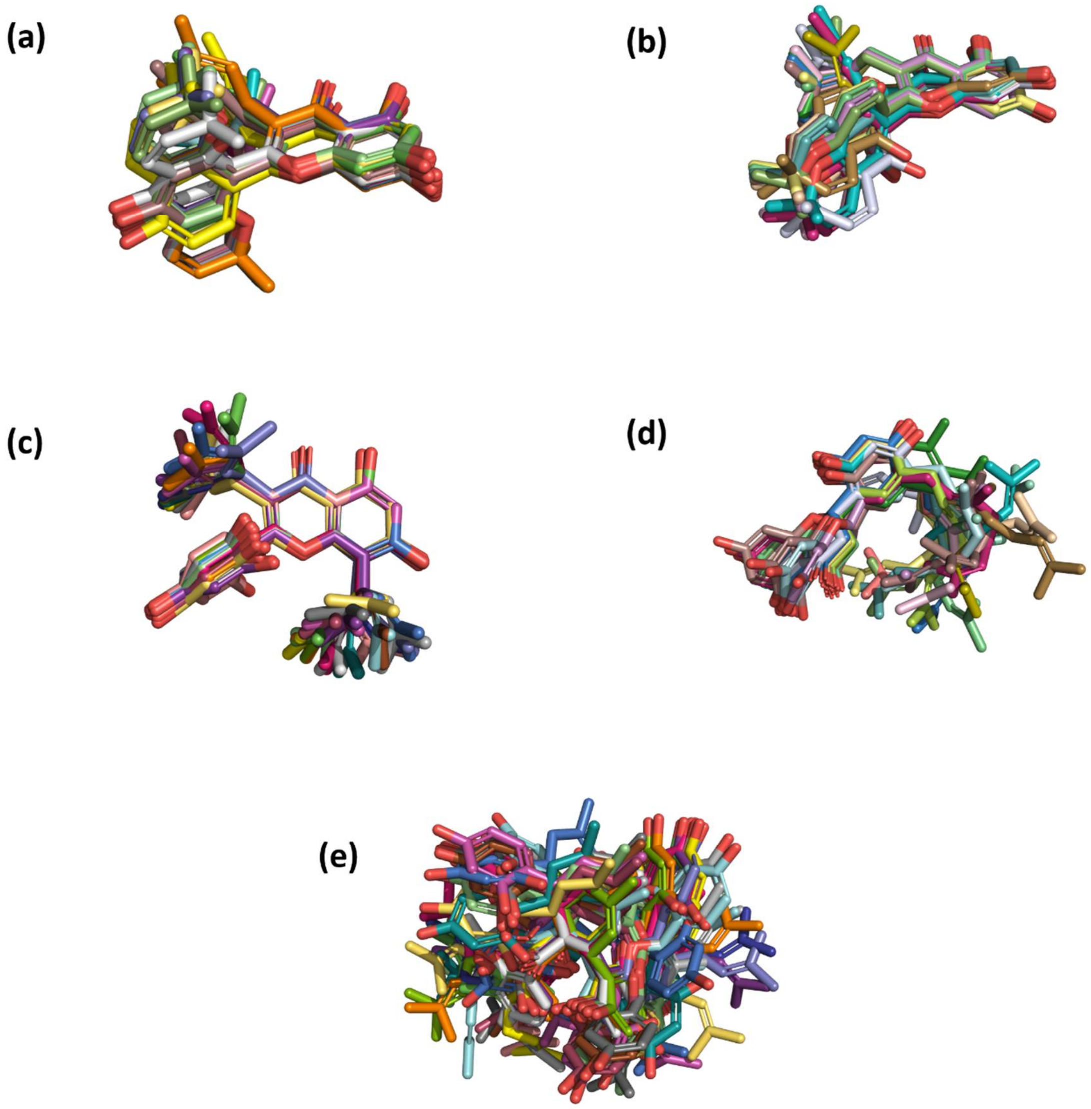

Figure S7. The superimposed structures for each binding pose of (a) Kuwanon A, (b) Kuwanon B, (c) Kuwanon C, (d) Kuwanon E, and (e) Kuwanon H. Based on the best binding pose of each kuwanon derivative, the average RMSDs among all binding poses were 9.303 Å in Kuwanon A, 10.651 Å in Kuwanon B, 7.313 Å in Kuwanon C, 7.956 Å in Kuwanon E, and 11.757 Å in Kuwanon H.

(a)

(b)

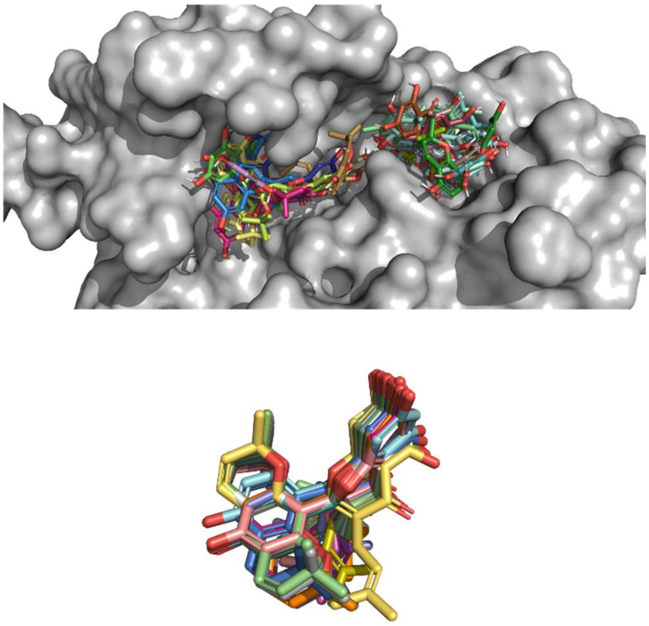

Figure S8. (a) All binding pose of Kuwanon A with generated 3D structure. (b) superimposed structures for each binding pose of Kuwanon A with generated 3D structure. Based on the best binding pose of each kuwanon derivative, the average RMSDs among all binding poses were 11.074 Å.

Supplementary Tables

Table S1. Docking score for Kuwanon derivatives and RMSD. Docking scores with RMSD greater than 10 Å were represented as a dash.

Rank	Kuwanon A		Kuwanon B		Kuwanon C		Kuwanon E		Kuwanon H	
	Score	RMSD	Score	RMSD	Score	RMSD	Score	RMSD	Score	RMSD
1	-8.9	-	-8.7	-	-7.6	-	-9.1	-	-8.6	-
2	-8.5	1.712	-8.7	1.716	-7.4	2.283	-9.0	3.478	-8.4	2.427
3	-8.3	5.245	-	-	-7.4	1.873	-8.9	3.576	-8.0	2.696
4	-8.3	3.625	-8.1	2.168	-7.2	4.033	-8.8	2.369	-	-
5	-8.1	5.242	-	-	-7.2	9.290	-8.4	2.856	-8.0	2.289
6	-7.9	3.184	-7.8	3.338	-7.2	2.206	-8.3	3.374	-	-
7	-7.7	4.113	-7.6	2.558	-7.2	9.100	-8.3	2.005	-7.7	9.984
8	-7.6	9.270	-7.6	3.859	-7.1	3.375	-8.2	3.828	-	-
9	-7.6	8.648	-7.6	2.980	-6.9	2.899	-8.1	4.154	-7.5	2.874
10	-7.5	4.116	-	-	-6.9	1.468	-8.1	1.557	-7.4	2.538
11	-7.4	3.300	-7.3	2.637	-6.9	3.260	-7.9	9.915	-7.2	8.163
12	-7.3	7.697	-7.3	8.050	-6.9	6.225	-	-	-	-
13	-	-	-7.0	3.604	-	-	-7.9	1.961	-7.1	7.595
14	-7.2	4.993	-6.9	8.032	-6.8	6.021	-7.8	3.485	-	-
15	-7.1	4.859	-	-	-6.7	1.408	-7.6	3.524	-7.0	2.532
16	-7.1	7.912	-6.8	4.208	-6.7	2.880	-	-	-6.9	5.792
17	-6.9	5.370	-6.8	9.122	-6.7	2.253	-7.6	4.016	-6.9	3.325
18	-6.8	5.008	-6.7	9.552	-6.6	2.846	-	-	-6.8	4.360
19	-6.8	4.579	-6.7	7.806	-6.6	1.523	-7.4	2.726	-6.8	4.776
20	-6.8	6.375	-6.6	3.097	-6.5	9.434	-	-	-6.8	3.367

Table S2. The total interaction energy between COX-2 and Kuwanon derivatives using FMO method. The underline means the binding pose with the lowest total interaction energy in each Kuwanon derivative. Total interaction energies with no value are binding poses far from Arg120 and Tyr355.

Index	Kuwanon A	Kuwanon B	Kuwanon C	Kuwanon E	Kuwanon H
1	-48.933	-52.663	-	-64.650	-77.101
2	-53.111	-55.210	-	-38.895	-58.176
3	-48.783	-	-	-66.969	-58.522
4	-50.993	-44.210	-60.355	-60.976	-
5	-49.969	-	-	-68.114	-54.963
6	-50.248	-47.807	-64.288	-	-
7	-34.786	-51.460	-	-48.157	-
8	-	-51.783	-42.785	-55.255	-
9	-	-47.462	-36.812	-45.463	-57.041
10	-42.323	-	-	-56.504	-65.856
11	-50.800	-43.972	-55.344	-	-
12	-	-	-44.969	-	-
13	-	-40.702	-	-48.108	-
14	-37.310	-	-	-59.039	-
15	-30.376	-	-	-41.942	-56.963
16	-	-43.725	-54.438	-	-
17	-	-	-	-36.522	-45.907
18	-37.424	-	-54.387	-	-54.110
19	-36.063	-	-	-39.969	-70.839
20	-38.664	-24.065	-	-	-

Table S3. The result of molecular docking simulation using generated and Pubchem 3D structure of Kuwanon A

Rank	Docking Score		RMSD[a]	
	Generated 3D structure	Pubchem 3D structure	Generated 3D structure	Pubchem 3D structure
1	-8.1	-8.9	-	-
2	-7.9	-8.5	1.766	1.712
3	-7.7	-8.3	1.744	5.245
4	-7.3	-8.3	2.679	3.625
5	-7.3	-8.1	12.159	5.242
6	-7.3	-7.9	1.657	3.184
7	-7.1	-7.7	14.378	4.113
8	-7.0	-7.6	14.441	9.270
9	-6.8	-7.6	2.168	8.648
10	-6.8	-7.5	12.876	4.116
11	-6.7	-7.4	13.596	3.300
12	-6.7	-7.3	18.613	7.697
13	-6.6	-7.3	2.167	12.097
14	-6.5	-7.2	15.014	4.993
15	-6.5	-7.1	2.950	4.859
16	-6.4	-7.1	17.483	7.912
17	-6.3	-6.9	17.298	5.370
18	-6.3	-6.8	15.977	5.008
19	-6.3	-6.8	11.647	4.579
20	-6.2	-6.8	15.472	6.375

[a]Root Mean Squared Distance (RMSD) was calculated between Rank 1 and other binding poses.
